# Effect of *Nigella sativa* on the kidney function in rats

**Published:** 2013

**Authors:** Mohammad Aziz Dollah, Saadat Parhizkar, Mohammad Izwan

**Affiliations:** 1 *Department of Biomedical, School of Medicine and Health Sciences, University Putra Malaysia *; 2 *Medicinal Plants Research Centre, Yasuj University of Medical Sciences (YUMS), Yasuj, Iran*

**Keywords:** Kidney, *Nigella sativa*, Rat, Toxicity

## Abstract

**Objectives:** *Nigella sativa* (*N. sativa*) is an amazing herb which is used in traditional medicine for a wide range of illnesses including bronchial asthma, dysentery, gastrointestinal problems, as well as beneficial effect on blood lipids, lowering blood pressure, serum cholesterol, and triglycerides level. This study aimed to determine the toxic effect of *N. sativa* powder on the kidney function which was evaluated by serum urea and creatinine and through histopathological examination of kidney tissue.

**Methods and Materials:** In this study, 24 male *Sprague Dawley* rats were randomly divided into four groups (six each). The rats were kept in the separate cage with three rats per cage. The treatment groups were given rat pellet containing *N. sativa *dose at 0.01, 0.10, and 1.00 g/kg body weight which were considered as low, normal, and high dose for five weeks while control group fed with rat chow pellet without supplementation. At the end of 35 days, the rats were sacrificed to take the blood sample and to remove the kidney organ for toxicity evaluation. Statistical analyses were done through one-way ANOVA using SPSS.

**Results:** The finding revealed that there was no significant difference in serum urea of treatment groups compared with the control group. The results showed a significant decline in serum creatinine of high dose of *Nigella sativa* treated compared with low dose treated and control groups (p<0.05). Histopathological examination of kidney tissue showed normal kidney architecture with no tissue degeneration, inflammation, necrosis, and tubular dilation in all groups.

**Conclusion:** With the evidence of normal urea and creatinine level in blood and normal kidney tissue in histology examination for all treatment groups, it is suggested that there is no toxic effect on kidney function of *Nigella sativa *at different doses for five-week period.

## Introduction

The use of natural products with therapeutic properties is as ancient as human civilization and for a long time, mineral, plant, and animal products were the main sources of drugs for therapeutic purpose (Hernandez-Ceruelos et al., 2002 ▶). Plants have always been a major source of nutrition and health care for both humans and animals. In recent years, there has been a growing interest in alternative therapies and the therapeutic use of natural products, especially those derived from plants (Schwartsmann et al., 2002 ▶). *N. sativa *(black seed*) *belongs to the *Ranunculaceae *family (El-Dakhakhny et al., 2000 ▶). It has been used as a herbal medicine for more than 2000 years. It is also used as a food additive and flavoring agent in many countries. The black seed oil is reported to be beneficial due to its content of over one hundred components such as aromatic oils, trace elements, and vitamins (Ali and Blunden, 2003 ▶). 

Recently, clinical and animal studies have shown that extract of the black seeds have many therapeutic effects such as immunomodulative (Boskabady et al., 2011 ▶; Hanafy and Hatem, 1991 ▶), antibacterial (Rakhshandeh et al., 2011 ▶; Zaoui et al., 2000 ▶), anti-tumor (Turkdogan et al., 2001 ▶), diuretic and hypotensive (Kanter et al., 2003), genoprotective (Babazadeh et al., 2012 ▶), hepatoprotective and antidiabetic (Houghton et al., 1995; Kanter et al., 2003) as well as bronchodilator activity (Boskabady and Sheiravi, 2002 ▶; Boskabady et al., 2004 ▶; Boskabady et al., 2010 ▶), and estrogenic activity (Parhizkar et al., 2011 ▶). Since 1970 to 2001, about 530 studies have been conducted on the *N. sativa *and only 3.4% concern about its toxicity *(*Anwar et al. 2005 ▶). One study shows that *N. sativa *fixed oil has a low toxicity with the evidence of high value of LD_50_ and no morphological changes on the histopathological examination on heart, liver, kidney, and pancreas tissue of treated rats (Al-Mofleh et al., 2008 ▶). Even though *N. sativa *is now commercially found in supplement, but the consumption in raw form is still popular. Usually, people consume *N. sativa *at a low dose because of unknown effect at high dose since there are no scientific data on the safety use of *N. sativa*. This study was carried out in order to determine the toxic effect of *N. sativa *seed consumption at various doses on the kidney function of rat. Kidney is the major organ in metabolizing toxic compounds besides liver. It receives about 1200 ml blood per minute (Tortora et al., 2006 ▶) containing a lot of chemical compounds and are at high risk to be exposed to toxic compounds. Therefore, high dose of *N. sativa *might cause damage to the kidney tissue which can be determined by measuring the level of urea and creatinine in blood as an indicator of kidney damage. The kidney damage also was determined by histological examination of kidney tissue.

Therefore, current study aimed to determine the toxic effect of *N. sativa *powder on the rats’ renal function which was evaluated by serum urea and creatinine and through histopathological examination of kidney tissue. 

## Methods and Materials

**Plant materials**

*N. sativa *seeds (imported from India) were purchased from a local herb store in Serdang, Malaysia. Voucher specimens of seeds were kept at the Cancer Research Laboratory of Institute of Biosciences and the seed was identified and authenticated by Professor Nordin Hj Lajis, Head of the Laboratory of Natural Products, Institute of Bioscience, Universiti Putra Malaysia. After cleaning the seeds under running tap water for 10 min, they were rinsed twice with distilled water and air dried in an oven at 40 °C overnight until a constant weight was attained. The seeds were ground to a powder shape using an electric grinder (National, Model MX-915, Kadoma, Osaka, Japan) for 6 min and were mixed with rat chow pellet powder and water into different doses including 0.01 (low dose), 0.1 (Normal dose), and 1 (High dose) g/kg of rats’ body weight. Afterward, dough was baked in an oven at 40 °C until it received instant weight. 

**Animals**

The protocol of the study was approved by Animal Care and Use Committee (ACUC), Faculty of Medicine and Health Sciences, Universiti Putra Malaysia (UPM) with UPM/FPSK/PADS/BR/UUH/F01-00220 reference number for notice of approval. Twenty-four male Sprague Dawley rats with 300-350 g body weight were supplied and cared in the Animal House of Faculty. The cages were kept in 29-32 °C temperature, 70-80% humidity, and automatic 12 hours light, 12 hours dark cycle which are suitable condition for Sprague Dawley rats. The animals were allowed to acclimatize for at least 10 days before the start of the experiments. The rats were fed with a standard rat chow and allowed to drink water *ad libitum. *All animals received human care and their handling was conducted between 08.00 and 10.00 am to minimize the effects of experimental stress. The treatments were given to the rats for 5 weeks. The body weight was measured once a week.

**Experimental design**

Animals were assigned into four treatment groups which are considered as control (0 g/kg b.w.), low (0.01 g/kg b.w.), normal (0.1 g/kg b.w.), and high (1.0 g/kg b.w.) doses of *N. sativa *for five weeks. Control group was given normal pellet without *N. sativa *while the other treatment groups were given pellet containing different doses of* N. sativa *dose respectively. The dosages were chosen based on human *N. sativa *consumption which is equal to 2 g/day and considering conversion rate to rats, 0.1 g/kg was selected as the normal dose. The low dose was ten times less than the normal dose while the high dose was one hundred times higher than the low dose. The treatments were given to the rats for five weeks.

**Statistical analysis**

Data were expressed as means±standard error of mean (SEM). The data were analyzed using SPSS windows program version 15 (SPSS Institute, Inc., Chicago, IL, USA). The one-way analysis of variance (ANOVA) and general linear model (GLM) followed by Duncan’s Multiple Range Test (DMRT) were used for analysis of data. A p-value less than 0.05 (p<0.05) was considered to be significant.

## Results

**Body weight**

**Figure 1:**
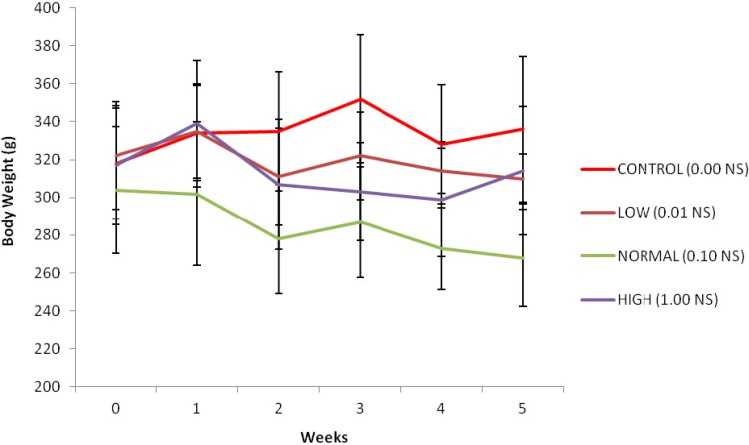
Changes in body weight of rats supplemented with various doses of *N. sativa* for five weeks.

Mean values for rats’ body weight supplemented with *N. sativa *powder at various doses for five weeks period was illustrated graphically in Figure 1. Measurement of the body weight was used to evaluate the health status of the rats during the treatment period. There was a significant difference in the body weights of rats from the start until the end of the treatment in all groups (p<0.05). 

**Serum creatinine**

**Figure 2:**
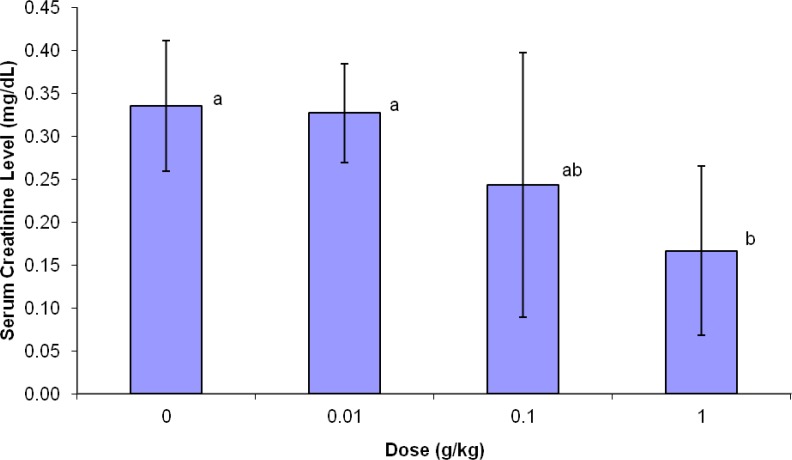
Changes in serum creatinine level of rats supplemented with various doses of *N. sativa *for five weeks.

The results shown in Figure 2 showed significant reduction (p<0.05) in serum creatinine of high dose *NS* (0.17±0.10) when compared with the control group (0.34±0.08) and low dose *N. sativa *(0.33±0.06) group.

Serum urea

The results of kidney function tests revealed reduction in serum urea concentration in normal dose *NS* group in comparison with the control group while, in low dose *NS* administrated group, the serum urea level was increased compared with the control group. On the other hand, there was a significant reduction (p<0.05) in serum urea concentration in normal dose *NS* (mean±SEM) in comparison with t he low dose *NS* group.

**Histopathological finding**

The histopathological examination showed normal architecture of the kidney of both the renal corpuscles and tubules of all rats as shown in Figure 4 (A, B, C, and D). In addition, the renal histological examination showed no tissue degeneration, inflammation, necrosis, and tubular dilation.

Data are expressed as mean±SEM. Treatments high dose=1 g/kg/day; Normal dose=0.1 g/kg/day); Low dose=0.01 g/kg/day *N. sativa *and 0 g/kg/day *N. sativa *supplemented with rat chow pellet.

Data are expressed as mean±SEM. Treatments high dose=1 g/kg/day; Normal dose=0.1 g/kg/day); Low dose=0.01g/kg/day *N. sativa* and 0 g/kg/day *N. sativa *supplemented with rat chow pellet.

**Figure 3:**
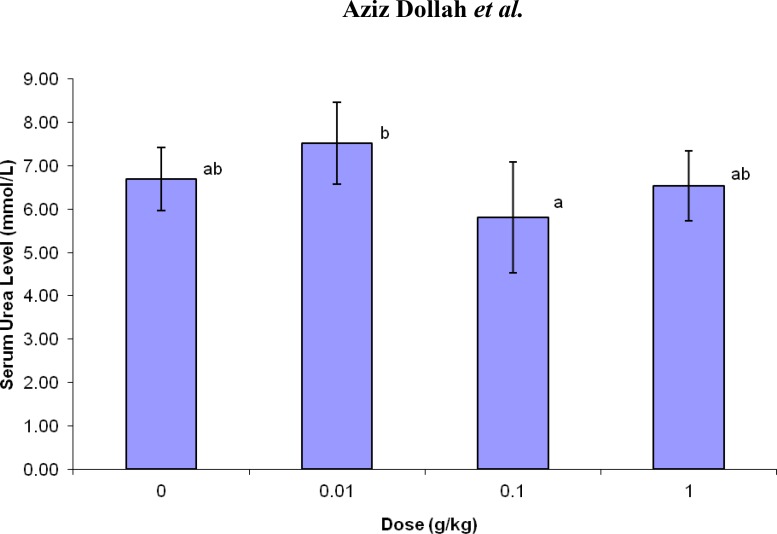
Changes in serum urea level of rats supplemented with various doses of *N. sativa *for five weeks.

Data are expressed as mean±SEM. Treatments high dose=1 g/kg/day; Normal dose=0.1 g/kg/day); Low dose=0.01 g/kg/day *N. sativa *and 0 g/kg/day *N. sativa *supplemented with rat chow pellet.

ab: Different superscripts indicated mean difference between groups significant at p<0.05.

**Figure 4:**
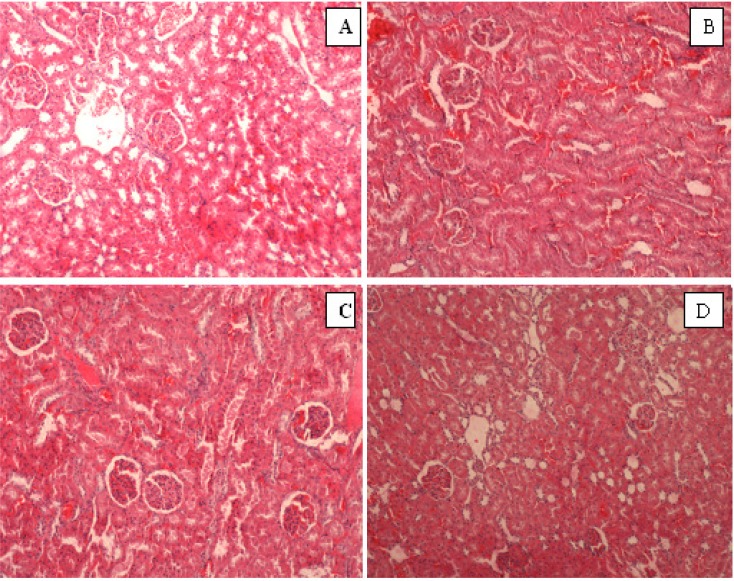
Histopathological section in the renal tissue of A) control group (0 g/kg *NS*), B) Supplemented with low dose NS (0.01 g/kg *NS*), C) Supplemented with normal dose (0. 1 g/kg *NS*), D) Supplemented with high dose *NS* (1 g/kg NS) (H&E, X100). The evaluation of kidney tissue revealed a normal structure of kidney similar to control group without any tissue degeneration, inflammation, necrosis, and tubular dilation

## Discussion

The present study was designed to investigate the toxicity effect of *N. sativa *on renal function by evaluation of the creatinine, blood urea, and histopathological changes of kidney. Urea is a byproduct from protein breakdown. About 90% of urea produced is excreted through the kidney (Walmsley et al., 2010 ▶). Meanwhile, the creatinine is a waste product from a muscle creatinine, which is used during muscle contraction. Creatinine is commonly measured as an index of glomerular function (Treasure, 2003 ▶). The normal range of serum creatinine is 0.2–0.8 mg/dl for rats (Weber et al., 2002 ▶). It is excreted exclusively through the kidney. Therefore, damage to the kidney will make the kidney inefficient to excrete both urea and creatinine and causes their accumulation in the blood. Therefore, the high level of blood urea and creatinine will indicate kidney damage. In our study, there was a significant reduction (p<0.05) in serum creatinine in the rats treated with high dose *N. sativa*.

The results of the present study showed that the supplementation of *N. sativa *to the diets of rats for five weeks did not change the biochemical parameters of kidney function as well as histopathological investigations which illustrated normal architecture of kidney. It’s proved by the presence of no significant change of serum urea of all treatment groups and creatinine level in low and normal doses groups compared with the control group. Absence of pathological condition of kidney tissue in histological evaluation confirmed our claim. This study also found that body weight of the rats in all groups were declined during the experiment, but the weight reduction of rats was not correlated to the toxicity of *N. sativa*, since no physical or behavioral signs of toxicity like lethargy, hyperactivity, restlessness, respiratory distress, or convulsions could be revealed. The same observations have been made by (Le et al., 2004 ▶) in normal rats treated with the petroleum ether extract of *N. sativa *for four weeks. Therefore, the rats maintained healthy and the results are not influenced by the health status of the rats. 

In accordance to our findings, it was previously proved that oral administration of aqueous extract of *N. sativa *seeds showed no significant changes in kidney function (Ali and Blundes, 2003 ▶). Another study also failed to show any toxicity for *N. sativa *fixed oil in mice (Zaouie et al., 2002 ▶; Alghamdi, 2003 ▶).

Our study showed that oral administration of *N. sativa *has no toxicity by different *NS* doses used. These results are in agreement with previous data reporting that *N. sativa *has a wide margin of safety (EL-Kholy et al., 2009 ▶; AL Ameen et al., 2011 ▶).

With the evidence of normal urea and creatinine level in blood and normal kidney tissue in histology examination for all treatment groups, it is suggested that there are no toxic effect on kidney function of *N. sativa *at different doses for five-week period. 

As a conclusion, the results of the present study showed the absence of toxic effect of *N. sativa* on rat kidney and suggest that popular consumption of *N. sativa* powder by human will not cause toxicity effect on the kidney function.
